# Hyperspectral Imaging Combined with Deep Transfer Learning to Evaluate Flavonoids Content in *Ginkgo biloba* Leaves

**DOI:** 10.3390/ijms25179584

**Published:** 2024-09-04

**Authors:** Jinkai Lu, Yanbing Jiang, Biao Jin, Chengming Sun, Li Wang

**Affiliations:** 1 College of Horticulture and Landscape Architecture, Yangzhou University, Yangzhou 225009, China; dx120200123@yzu.edu.cn (J.L.); 13205227999@163.com (Y.J.); bjin@yzu.edu.cn (B.J.); 2 Jiangsu Key Laboratory of Crop Genetics and Physiology/Co-Innovation Center for Modern Production Technology of Grain Crops, College of Agriculture, Yangzhou University, Yangzhou 225009, China; cmsun@yzu.edu.cn

**Keywords:** *Ginkgo biloba*, flavonoids, hyperspectral imagining, deep learning

## Abstract

*Ginkgo biloba* is a famous economic tree. Ginkgo leaves have been utilized as raw materials for medicines and health products due to their rich active ingredient composition, especially flavonoids. Since the routine measurement of total flavones is time-consuming and destructive, rapid, non-destructive detection of total flavones in ginkgo leaves is of significant importance to producers and consumers. Hyperspectral imaging technology is a rapid and non-destructive technique for determining the total flavonoid content. In this study, we discuss five modeling methods, and three spectral preprocessing methods are discussed. Bayesian Ridge (BR) and multiplicative scatter correction (MCS) were selected as the best model and the best pretreatment method, respectively. The spectral prediction results based on the BR + MCS treatment were very accurate (R_Test_^2^ = 0.87; RMSE_Test_ = 1.03 mg/g), showing a high correlation with the analytical measurements. In addition, we also found that the more and deeper the leaf cracks, the higher the flavonoid content, which helps to evaluate leaf quality more quickly and easily. In short, hyperspectral imaging is an effective technique for rapid and accurate determination of total flavonoids in ginkgo leaves and has great potential for developing an online quality detection system for ginkgo leaves.

## 1. Introduction

*Ginkgo biloba* L. is a very ancient species, known as a living fossil in the plant kingdom [1]. In addition, ginkgo is also an extremely important and famous economic species, and its leaves have been cultivated for pharmaceutical and industrial products, which are widely produced around the world [2,3]. The *G. biloba* leaf is rich in flavonoids, which can modulate the levels of reactive oxygen species (ROS), thereby helping to maintain redox homeostasis and prevent DNA damage [4]. For instance, under the stress of ultraviolet B (UV-B) radiation, the accumulation of flavonoids can serve as a “sunscreen” to protect cells from radiation-induced damage [5]. Due to the rich content of flavonoids in its leaves, it has become a raw material for various medicinal products [6]. The extract of *G. biloba* leaves (GBE) is commonly utilized for the treatment of cardiovascular diseases frequently encountered in the elderly population. The core constituents of GBE are flavonoid compounds, and the medicinal extract derived from dried *G. biloba* leaves is typically standardized to contain 24% flavonoids [7].

Quality attributes determine the commercial value of *G. biloba*. Generally, the higher the content of medicinal ingredients in ginkgo leaves, the higher the economic value. The production of flavonoid compounds in ginkgo is influenced by many factors, including the selected variety [8,9], harvest time [9], environmental conditions [4,5,10], and the extraction process [11,12]. In addition, the flavonoid content is also affected by the development status, and the older the plants, the lower the flavonoid content. Under normal circumstances, ginkgo leaves that are more than 5 years old do not have medicinal extraction value due to their low content of effective ingredients [13]. Therefore, the quantitative determination of flavonoid compound concentration in ginkgo leaves has important significance for quality sorting and consumption. However, conventional methods for determining the flavonoid content, such as spectrophotometry and high-performance liquid chromatography (HPLC), are destructive, with low efficiency, prolonged sampling time, strong dependence on reagents, and high cost. They are also only suitable for small batches of samples and require personnel with specialized experimental knowledge and skills, which under normal circumstances makes it difficult to meet the detection requirements. Therefore, there is an urgent need for online monitoring of the flavonoid content in *G. biloba* leaves to address the issues of rapid, accurate, and non-destructive measurement of the flavonoid content.

Machine vision technology employs optical devices to capture real-world imagery, subsequently processing and analyzing the image information using computers to perform preset operations on required information or control mechanical execution devices. This constitutes a non-contact measurement technique. Currently, this technology has become one of the most commonly used and efficient methods for non-destructive quality inspection of leaves [14,15]. It can combine leaf images with machine learning algorithms to classify leaves of different qualities by analyzing the differences in phenotypic characteristics such as size, shape, and texture. However, it is challenging to apply this method to leaves with similar phenotypes [16]. Fortunately, high-throughput phenotyping analysis-high spectral imaging (HSI) can overcome this issue by integrating the multispectral information of the leaves. As a rapid, accurate, and non-destructive technique, HSI has been widely applied in the detection of various agricultural products to overcome phenotypic bottlenecks in large-scale production [17]. For instance, this technology has been utilized to differentiate the characteristics of fresh tea leaves to determine the optimal harvest time [15]. Additionally, the estimation of chlorophyll levels in wheat using HSI allows for the rapid screening of nitrogen responses in large wheat populations [17]. Furthermore, this method can effectively distinguish and classify target objects or predict crop traits by detecting subtle differences in the chemical composition and distribution within the leaves [18], such as anthocyanin content in purple-fleshed sweet potatoes [19], the soluble solid content of apples [20], the total flavonoids content (TFC) in okra (*Abelmoschus esculentus* L.) pods [21], the total polysaccharides and total flavonoids in *Chrysanthemum morifolium* [22], and so on. Furthermore, HSI technology has been used for rapid, non-destructive testing and visualization of the quality of some medicinal plants. In previous work, HSI has been combined with regression analysis for estimating the concentration of tetrahydrocannabinolic acid [23] and cannabidiolic acid in hemp (*Cannabis sativa* L.) [24]. The above research shows the feasibility of using HIS technology to predict the quality of agro-products. However, to our knowledge, there has been limited research on the use of HSI technology to determine and visualize the total flavonoid content of ginkgo leaves.

However, the combination of hyperspectral technology with multivariate analysis still faces several challenges in practical applications. For instance, the accuracy and robustness of different algorithms in detecting the chemical composition of plant leaves using high-resolution images can vary between training and validation datasets. Additionally, the generalization capabilities of these algorithms in the resolution domain may also differ [21,23]. In particular, the acquired spectral data are influenced by a multitude of external factors, such as noise in the detection environment, variations in the chemical and physical properties of the samples, and measurement errors of the instruments [25]. The spectral preprocessing methods can effectively address these issues. Spectral preprocessing can reduce or eliminate the impact of various non-target factors, ensuring the universality and effectiveness of spectral data, and enhancing the predictive power and robustness of the models [26,27]. Specifically, different preprocessing methods can lead to varying performance of spectral models. Generally speaking, the optimal choice of spectral preprocessing is empirical and exploratory [28]. Therefore, it is crucial to select the appropriate preprocessing method to improve the accuracy of the model.

Deep transfer learning is an emerging subfield within the domain of machine learning, renowned for its capability to handle extremely large-scale datasets and its exceptional generalization performance on unseen data. However, due to the scarcity of relevant datasets, there have been few reports on the assessment of flavonoid content in Ginkgo biloba leaves using deep learning and hyperspectral imaging technologies. In this study, we explore the feasibility of utilizing hyperspectral imaging (HSI) technology for rapid, non-destructive prediction and visualization of total flavonoid content in ginkgo leaves. Initially, we acquire hyperspectral images of 380 sets of *G. biloba* leaf samples across the 400~1000 nm wavelength range, alongside reference values for total flavonoids, and extract the average spectral data from the region of interest (ROI). The spectral data are then subjected to multiplicative scatter correction (MSC), standard normal variate transformation (SNV), and Savitzky–Golay (SavGol) preprocessing to refine the spectral characteristics. Feature variables are extracted using the OTSU algorithm. Ultimately, the dataset was randomly partitioned into a 4:1 ratio, resulting in a training set comprising 304 samples and a testing set with 76 samples. Six predictive models were established and their accuracy was evaluated to determine the optimal model for predicting the total flavonoid content in leaves.

## 2. Results

### 2.1. Differences in Ginkgo Leaf Shapes and Total Flavonoid Content

**Figure 1:**
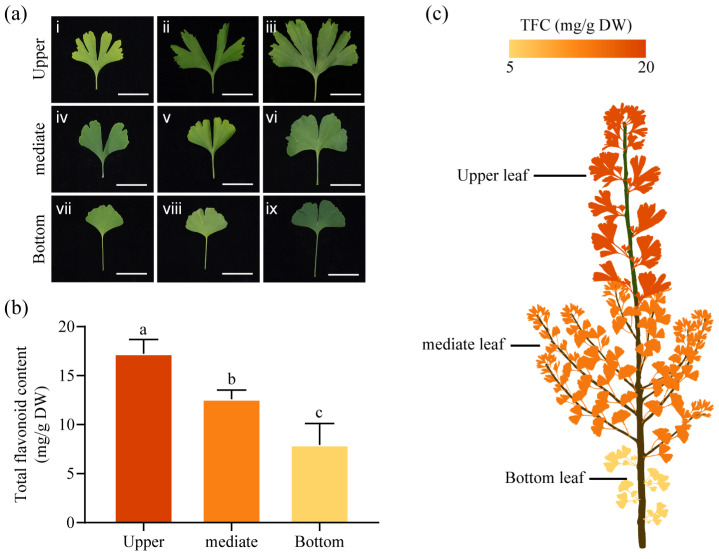
An analysis of total flavonoid content (TFC) was conducted on ginkgo leaves of different morphological regions. (**a**) Morphologies of the upper, middle, and lower parts of the leaves on a 5-year-old ginkgo tree. Among them, vii and viii represent unlobed leaves (UL), and i, ii, iii, iv, v, vi, and ix represent lobed leaves (LL). scale bar = 5 cm. (**b**) TFC in leaves from different regions. Data are presented as the mean ± standard deviation (SD) of three independent biological replicates. Different letters indicate significant differences determined by Tukey’s Honestly Significant Difference (HSD) test: *p* < 0.05. (**c**) A heatmap represents the differences in total flavonoid content among various regions of the ginkgo tree. The deeper the color, the higher the TFC.

We randomly selected a ginkgo tree and found that the leaves in different parts had different morphological characteristics (Figure 1a). We divided them into nine types according to the leaf cleft and edge angle. Further observation showed that the upper leaves of the trunk usually had multiple leaf cracks, while the middle leaves had less leaf fission and the lower leaves had the fewest leaf cracks. The content of total flavonoids in leaves of different parts of the upper, middle, and lower parts of the trunk was the highest, followed by the middle, and then the lowest (Figure 1b,c). These results indicated that the content of total flavonoids in ginkgo leaves was related to leaf phenotype and leaf location.

### 2.2. Flavonoid Composition Determination of Ginkgo Leaves and Sample Set for HIS

**Figure 2:**
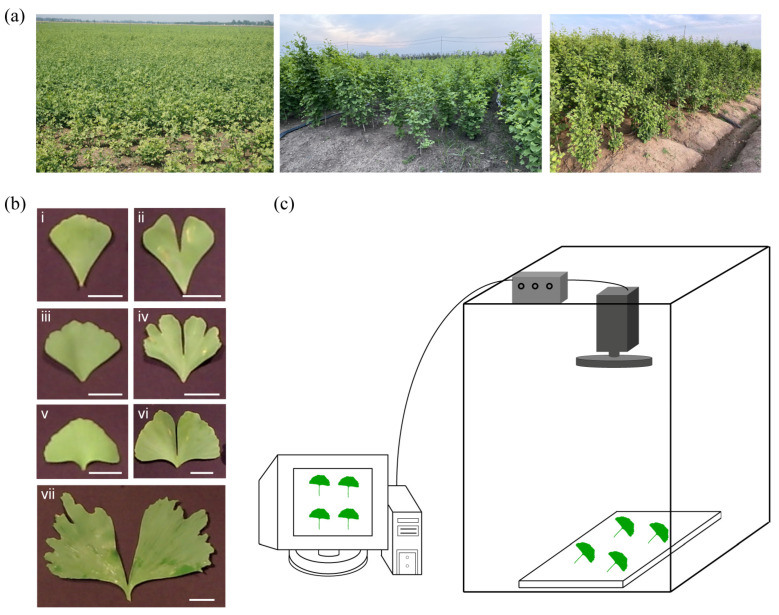
Flowchart of the hyperspectral data acquisition process for ginkgo leaves. (**a**) Sampling site for ginkgo leaf samples. (**b**) Morphological characteristics of some ginkgo leaf samples. Among them, i, iii, and v represent UL, and ii, iv, vi, and vii represent LL. scale bar = 5 cm. (**c**) Hyperspectral imaging system.

**Figure 3:**
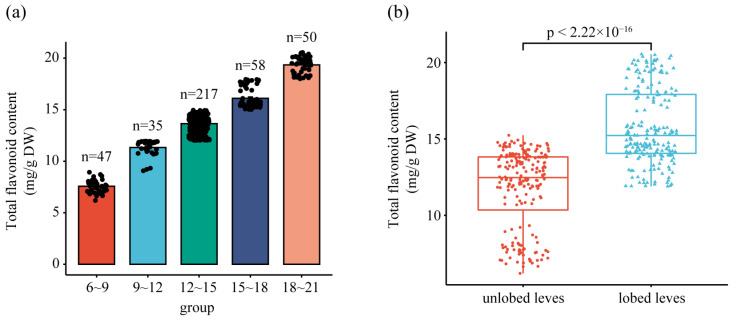
Statistical results of the TFC for all samples. (**a**) Distribution statistics of the TFC. (**b**) Statistics of the TFC in leaves with and without lobes.

Since the TFC of ginkgo leaves was related to leaf phenotype, 407 groups of ginkgo leaves from 1 to 5 years old were randomly sampled, and their spectral information was acquired using a hyperspectral imaging system (Figure 2). The statistical results of TFC in 407 groups of ginkgo leaf samples by spectrophotometry are presented in Figure 3. The TFC values range from 6.1969 mg·g^−1^ to 20.5526 mg·g^−1^, which are comparable to the data in previous studies, proving that establishing a TFC prediction model on this dataset is effective [13,29]. Furthermore, the quantity distribution of flavonoids in the samples was mostly concentrated at 12~15 mg·g^−1^ (Figure 3a), which is consistent with the typical flavonoid content found in leaves from 1 to 5 years of age [13]. Especially, there is a certain correlation between the leaf lobe phenotype and the flavonoid content of the leaves. We found that the TFC was higher in leaves with lobed leaves than in leaves without lobed leaves (Figure 3b). This could be particularly useful if the producer decides to harvest leaves with a higher flavonoid content. Leaf splitting is an important phenotype of leaf age, and in general, younger leaves have more and deeper leaf splitting. Previous studies have reported a greater accumulation of flavonoids in young ginkgo leaves compared to older leaves [29]. Therefore, we speculate that the effect of leaf splitting on flavonoids was mainly affected by plant age.

### 2.3. Spectra Characteristics of Ginkgo Leaves

**Figure 4:**
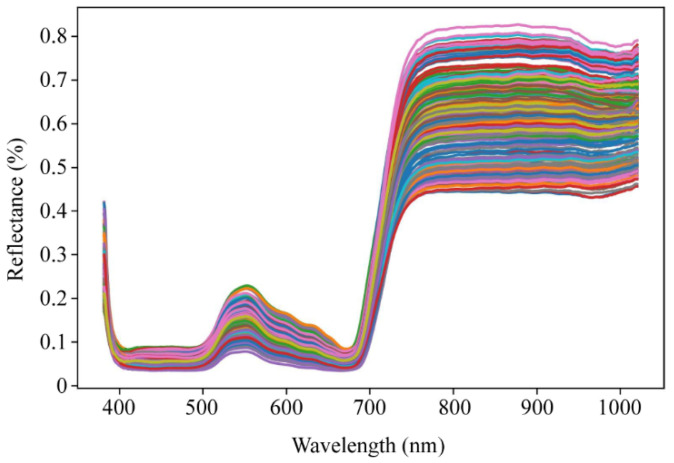
The reflectance spectrum of ginkgo leaf samples.

The average reflectance spectra of all samples are depicted in Figure 4. Across the entire visible to near-infrared spectral data ranging from 381.8 nanometers to 1020.5 nanometers, there are some intersections and overlaps between the spectra of different samples, yet all spectra exhibit similar trends. The vibrations observed in the visible light region around the 500~700 nanometer band are attributed to chlorophyll, which is associated with the nitrogen content in the leaves of green plants. The rapid changes in reflectance within the 680~750 nm range are indicative of the well-known “red edge” of plants in the electromagnetic spectrum [30]. Furthermore, minor weak peaks observed in the range of 750 nm to 930 nm are predominantly attributed to the third overtone stretching of the O-H functional groups associated with water in the ginkgo leaf samples. An absorption region observed in the 930~1000 nm range corresponds to the third harmonic of the C-H functional groups and the second overtone of the O-H harmonics [31]. The variations in the spectral characteristics of ginkgo leaves suggest that hyperspectral imaging holds potential for predicting flavonoid content.

### 2.4. Analysis of Different Models

In this study, we employed deep learning for regression analysis. To train a deep learning model, a training set and a test set are required. Spectral outliers were identified using the Hotelling T2 test. Twenty samples were identified as spectral outliers and excluded from the sample set, resulting in a final set of 380 samples that were matched between the corrected hyperspectral data and the flavonoid data. The 380 samples were randomly divided into a training set and a test set in a 4:1 ratio. Subsequently, six different models for total flavonoid content (TFC) analysis (Support Vector Regression, SVR; Partial Least Squares Regression, PLSR; Bayesian Ridge Regression, BR; Linear Regression; Lasso Regression; and Ridge Regression) were established based on the previous data. These models were designed to capture the relationship between the ginkgo leaf spectral data and the TFC values by inputting the entire spectral matrix and the TFC values. To mitigate the likelihood of model overfitting, the GridSearch method was applied to the training set during the model construction process.

**Figure 5:**
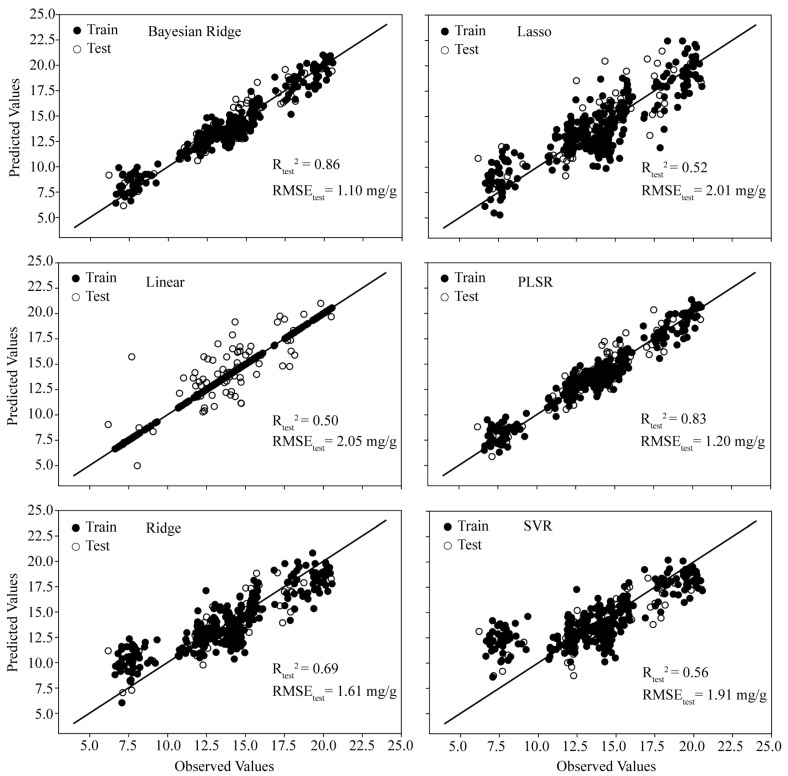
The training and testing results for estimating the TFC in ginkgo leaves using six different algorithms. The training set is represented by solid circles, and the test set is represented by hollow circles. R_Test_^2^ (coefficient of determination for test); RMSE_Test_ (root mean square error for test).

The relationship between the measured and the modeled leaf flavonoid content is shown in Figure 5. The regression results showed that the PLSR model and the BR model are better among the eight models. The cross-validated R_Test_^2^ of the PLSR model was 0.83 with an RMSE_Test_ of 1.2 mg·g^−1^. The BR model outperformed the PLSR model, with R_Test_^2^ and RMSE_Test_ of 0.86 and 1.10 mg·g^−1^, respectively. The results indicate that establishing a predictive model for the total flavonoid content (TFC) in ginkgo leaves based on visible and near-infrared spectroscopy is feasible. The superior performance of the linear model may be attributed to the predominance of linear patterns in the relationship between the spectra and the TFC.

### 2.5. Effects of Different Pretreatments on Model Transfer

**Figure 6:**
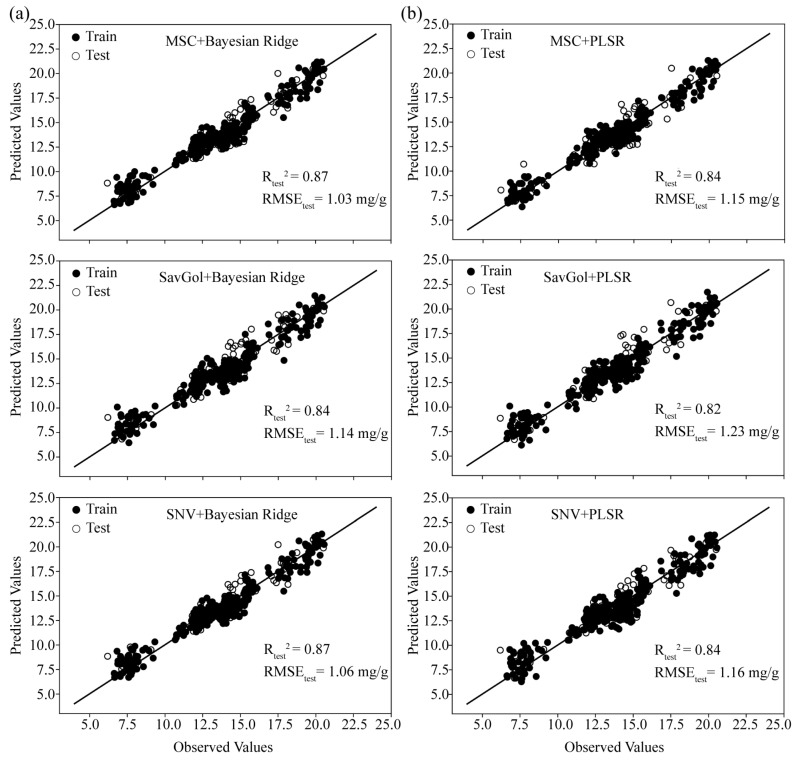
The results of the Bayesian Ridge (**a**) model and the PLSR model (**b**) established based on the multiplicative scatter correction (MSC), standard normal variate transformation (SNV), and Savitzky–Golay (SavGol) preprocessed spectra. The training set is represented by solid circles, and the test set is represented by hollow circles.

To select the optimal preprocessing method, the preprocessed spectral data were used as input variables in the PLSR and BR models to establish the corresponding prediction model (Figure 6). Three pretreatment methods were employed: MSC, SNV, and SavGol. Compared with the results of raw spectral data, the spectral pretreatment methods of MSC and SNV can improve the prediction performance of PLSR and BR models, while SavGol reduces the predictive performance of PLSR and BR models. Furthermore, the PLSR model using the preprocessing method of MSC showed more accuracy than other models, with R_Test_^2^ increasing by 1.20% and RMSE_Test_ declining by 4.17% compared to models built with original spectra. Additionally, the BR model combined with MSC preprocessing showed better performance than the BR models combined with SNV, with R_Test_^2^ increasing by 1.15% and RMSE_Test_ declining by 6.36% based on the raw spectra modeling (Figure 6). Based on the aforementioned analysis, MSC was chosen as the optimal preprocessing method, demonstrating strong generalization capability.

## 3. Discussion

Flavonoids are the most important secondary metabolites in *Ginkgo biloba*, and the content of flavonoids determines the value of ginkgo leaves [32]. The traditional methods for the determination of flavonoids are ultraviolet spectrophotometers and liquid mass spectrometry, which makes the determination method time-consuming and difficult to perform in the field. Recently, hyperspectral technology and deep learning have been widely used as fast and efficient methods to predict the physiological parameters of plants [33]. In particular, the prediction of active ingredients in some plants has good performance, such as the prediction of tea composition [34]. Furthermore, in okra pods, Cui et al. [21] reported R^2^ over 0.93 for TFC. In black goji berries, Zhang et al. [35] reported R^2^ over 0.95, 0.91, and 0.93 for total flavonoids, total anthocyanins, and total phenolics, respectively. For cannabidiolic acid, Ooi [24] reported an average R^2^ value of 0.98 in *Cannabis sativa* L. The prediction performances of total anthocyanins, total flavonoids, and total phenolics exhibit variability across different studies. In this study, we collected a large number of ginkgo leaf samples and measured the flavonoid contents. The estimation of flavonoid contents with deep learning was accomplished.

However, the integration of hyperspectral technology with multivariate analysis encounters several issues in practical applications. The obtained spectra are influenced by a variety of factors, such as noise in the measurement environment, differences in the chemical and physical properties of the samples, and instrumental measurement errors [25]. These issues can make it difficult for models built on the previous set of samples to be used for the next set of samples. Previously established models may also be difficult to apply to other varieties or after changing measurement conditions [36]. Spectral preprocessing can eliminate background information and noise, mitigate the effects of various factors, and preserve the useful information of the samples to the greatest extent possible. Furthermore, preprocessing can also enhance the versatility of the spectrum, improve the prediction error of the model, and improve its robustness. Therefore, spectral preprocessing is necessary to establish a reliable and stable model [37]. The common preprocessing methods include SavGol, MSC, SNV, variable sorting for normalization (VSN), and first-derivative (FD) methods [38]. Xiao et al. [25] discussed seven different spectral preprocessing methods and found that the model built by combining FD + SNV preprocessing with deep transfer learning was superior to the conventional model. In the study of the identification and classification of sea cucumbers by hyperspectral technology, it was found that VSN and SNV are the best preprocessing methods for filtering out noise and scatter information in the raw spectra, which can enhance the model’s recognition performance [39]. In general, a good model has two aspects: a higher R^2^ and a lower RMSE value. In this study, we found that after MSC and SNV pretreatment, the R^2^ increased and the RMSE decreased, indicating that the accuracy of the model was improved. Similarly, in studies of capsaicin content determination, it has been found that MSC and SNV pretreatments eliminate or reduce the effects of spectral scattering, thereby improving the precision of the model [40]. In contrast, SavGol pretreatment technology did not show the same robustness as other preprocessing methods and even performed worse than unpreprocessed data. Similarly, a recent study assessing the adulteration of sesame oil also found that SavGol pretreatment methods reduce the accuracy of the model [41]. Therefore, the selection of an appropriate preprocessing method is crucial for enhancing the robustness of the model.

In addition, the findings of this study also found that the TFC of ginkgo leaves was highly correlated with leaf cracks. The flavonoid content of leaves with leaf clefts was higher than that of leaves without leaf clefts. We speculate that this may be related to the age of the leaves. Previous research has identified age as a significant factor influencing the synthesis and accumulation of flavonoid compounds. In comparison, the flavonoid content in the young leaves of hawthorn and birch is markedly higher than that in mature leaves [42,43]. Similarly, young leaves of ginkgo trees possess high levels of flavonoids, whereas their content significantly decreases in mature trees [7]. Young leaves often have a deep and numerous leaf-splitting phenotype [29,44]. This result can help leaf harvesters evaluate the flavonoid content of leaves more intuitively and quickly.

## 4. Materials and Methods

### 4.1. Sample Preparation

An experiment was conducted in August 2021 at the leaf-use ginkgo nursery located in Pizhou City, Jiangsu Province, China (34° 36′ 27′′ N, 117° 58′ 47′′ E). A total of 407 groups of *G. biloba* (‘Fozhi’) leaf samples from 1 to 5 years old were collected and divided into two categories, including lobed leaves (LL, 207 samples) and unlobed leaves (UL, 200 samples). All freshly harvested *G. biloba* leaves were individually placed on a blackboard for the acquisition of hyperspectral imaging data. Following the data collection, the leaves were dried and ground into a fine powder for the determination of TFC.

### 4.2. Spectra Acquisition

Utilizing a near-infrared hyperspectral imaging system, hyperspectral imaging was conducted on fresh ginkgo leaves. The hyperspectral imaging system consists of a darkroom, a hyperspectral spectrometer, and a computer. The core component of the system was a stepping motor-driven moving platform designed for the linear scanning of samples, complemented by two symmetrically positioned halogen lamps that provide a stable light source. Prior to the acquisition of leaf spectral data, a white reference image was first obtained (using a standard whiteboard with a reflectance close to 100%) and a dark reference image was acquired (by turning off the light source and completely covering the camera lens with an opaque cap), to correct the raw intensity images to reflectance images. The corrected images are calculated using the original hyperspectral images, the white reference image, and the dark reference image. The grayscale correction formula is depicted as follows:

$$I=\frac{I_{raw}-I_{b}}{I_{w\;}-{\;I}_{b}}$$

In the formula, *I* represents the corrected image data, *I_raw_* signifies the raw image data, *I_b_* denotes the dark background image data obtained when the device cover seals the camera, and *I_w_* refers to the whiteboard image data captured when a whiteboard is placed in a position corresponding to the object under test, filling the frame acquisition range of the hyperspectral camera.

### 4.3. Spectral Extraction

The data processing of hyperspectral imaging is relatively more complex, with the specific technical procedure as follows:

- Initially, a visible-light image was extracted from each hyperspectral image.
- From the visible light image, a gray-scale image was extracted, and an EGI (Enhanced Greenness Index) image was obtained through the 2G-R-B operation. Otsu’s algorithm was employed to automatically determine the threshold for segmenting the gray-scale image, yielding the gray mask (i.e., the locational region of the black cloth within the image). Similarly, Otsu’s algorithm was used to ascertain the threshold for segmenting the EGI image, resulting in the EGI mask (the locational region within the yellow-green color spectrum of the image). The intersection of these two locational regions was taken to obtain the mask for the leaf’s position.
- Based on the mask position information of the foreground, the hyperspectral image calculated the hyperspectral mean for each pixel point of the leaf, thus obtaining the spectral information for each leaf. This spectral information was then supplemented with corresponding flavonoid data to produce the final dataset used for modeling.

### 4.4. Measurements of Flavonoids Content

After acquiring hyperspectral imagery, an immediate assessment of the TFC in the ginkgo leaves was conducted. The ginkgo leaf samples were dried to a constant weight in an oven, then ground into a powder and passed through an 80-mesh sieve. A precise amount of 0.02 g of the powdered sample was weighed into the centrifuge tube, and 2 mL of extraction solvent (60% ethanol) was added, achieving a material-to-solvent ratio of 1:100. The samples were extracted under vortex agitation conditions at 60 °C for 2 h. Subsequently, the mixture was centrifuged at 12,000 rpm at 25 °C for 10 min, and the supernatant was collected for determination. Using a pipette, 540 μL of the supernatant was transferred into a 2 mL centrifuge tube. Following the method outlined in the kit’s instruction manual (Suzhou Comin Biotechnology Co., Suzhou, China), reagents were sequentially added to the supernatant, the mixture was vortexed, and then allowed to stand in the dark for 15 min. In the end, the TFC was calculated by measuring the absorbance at 510 nm using a UV-Vis spectrophotometer.

### 4.5. Spectral Preprocessing

In the acquisition of spectral data, there is a frequent occurrence of disturbances due to extrinsic environmental factors and instrumental responses that are extraneous to the intrinsic properties of the samples under examination. To confer superior predictive capabilities upon the models developed, it becomes necessary to apply suitable methodologies for the preprocessing of spectral datasets. Within the purview of this research, a triad of preprocessing algorithms has been adopted, encompassing MSC, SNV, and the application of SavGol.

MSC is a commonly used algorithm in multi-band calibration modeling, which can eliminate spectral differences caused by different scattering conditions to a certain extent and enhance the correlation between features and predicted data. MSC initially calculates the mean of all sample features, then performs a univariate linear operation between each sample’s features and the mean to determine the translation and offset of each sample relative to the mean of all features. By eliminating the translation in the original features of each sample and dividing by the offset, the corrected spectral feature data can be obtained [45]. The specific calculation formulas are as follows:

- Average spectral data:
  $$\vec{x_{i}}=\frac{1}{n}\sum_{i=1}^{n}x_{i}$$
- Univariate linear operation:
  $$x_{inew}=k_{i}\vec{x_{i}}+b_{i}$$
- Corrected spectrum:
  $$x_{i\left(MSC\right)}=\frac{x_{inew}-b_{i}}{k_{i}}$$

In the formulas a, b, and c: *x_i_* represents the spectral data of an individual sample, and *k_i_* and *b_i_* denote the offset and intercept, respectively, obtained after performing a simple linear regression between the spectral data of each sample and the average spectrum.

SNV is commonly utilized to mitigate adverse factors such as shape asymmetry and non-specific scattering on the target surface. This method is applied to process the characteristics of individual samples [45,46]. The specific calculation formula is as follows:

$$x_{i,SNV}=\frac{x_{i}-\overset{-}{x}}{\sqrt{\frac{\sum_{i=1}^{k}{\left(x_{i}-\overset{-}{x}\right)}^{2}}{k-1}}}$$

In the formula, *I_x_* represents the original spectral reflectance of the individual sample, $\overset{-}{x}$ denotes the average spectral reflectance of the samples, and *k* signifies the number of spectral bands.

The SavGol method is an algorithm for least squares convolution fitting that preserves distributional characteristics such as relative maxima, minima, and widths. Its application to the preprocessing of spectral data can enhance the smoothness of the data. The computational process is as follows:

- Determine a window of fixed size (2m + 1), considering all data within this window as a collective set.
- For each measurement point *x* = [−m, 1 − m, …, −1, 0, 1, …, m], the following formula is employed for fitting:
  $$p\left(x\right)=\sum_{k=0}^{n}{a_{k}x}^{k}$$
- By calculating the least squares residuals between the fitted curve and the spectrum and setting them to the minimum as boundary conditions, the optimal coefficient matrix B can be obtained through the computation B = X(X^T^X)^−1^X^T^. The convolution of B with the sample spectrum is then performed to achieve SavGol filtering.

### 4.6. Model Establishment

The SVR model is an application of the Support Vector Machine (SVM) to regression problems, capable of effectively handling high-dimensional data and providing robust performance even when the feature space is substantially large. By incorporating the kernel trick, SVR can address nonlinear issues and exhibit high robustness against noise and outliers. Furthermore, SVR adheres to the principle of maximum margin characteristic of SVM, which contributes to enhancing the model’s generalization ability and preventing overfitting.

In this study, in addition to the SVR model, we also established and compared the predictive accuracy of five other models (including PLSR, BR, Linear, Lasso, and Ridge) to determine the optimal model for estimating the TFC in ginkgo leaves.

### 4.7. Model Evaluation

The performance of a model is assessed through the determination coefficient (R^2^) and the root mean square error of the training set (RMSE). For the models, R^2^ represents the percentage of variance in the predicted values of the target variable explained by the model. In general, an R^2^ value that falls within the interval of 0.61 to 0.8 is indicative of a model that is deemed suitable for predictive purposes. An R^2^ value positioned between 0.81 and 0.9 denotes a model that exhibits commendable performance, whereas an R^2^ value surpassing 0.9 signifies a model with an exceptionally high predictive efficacy [47]. RMSE is utilized to measure the deviation between the model’s predicted values and the actual measurements. The lower the RMSE value, the more accurate the predictive performance of the model. The calculation formulas for R^2^ and RMSE are as follows:

$$R^{2}=1-\frac{\sum_{i=1}^{n}{\left(y_{i}-\hat{y_{i}}\right)}^{2}}{\sum_{i=1}^{n}{\left(y_{i}-y_{m}\right)}^{2}}$$

$$\mathrm{RMSE}\;=\sqrt{\frac{1}{n}\sum_{i=1}^{n}{\left(y_{i}-\hat{y_{i}}\right)}^{2}}$$

In these formulas, *y_i_* and $\hat{y_{i}}$ denote the actual and predicted values of the measured indicator in the calibration and prediction sets, respectively, while *y_m_* represents the mean value of the measured indicator within the dataset.

## 5. Conclusions

In this study, we explored the possibility of combining hyperspectral techniques with deep learning algorithms for the detection of total flavone content in ginkgo. For the prediction of total flavonoids in ginkgo leaves, it demonstrated the highest predictive accuracy. Furthermore, we compared the impact of different preprocessing techniques on the model’s precision. It can be observed that different preprocessing methods influence the accuracy of the BR model. In summary, MSC outperforms other preprocessing techniques. The model that combines spectral preprocessing with deep transfer learning has achieved satisfactory results, thereby validating the effectiveness of the proposed approach. In future research, a broader range of *G. biloba* varieties and spectral variations will be considered to further enhance the robustness of the model.

## Data Availability

The data that support the findings of this study are available on request from the corresponding author.
